# Post-and-Core Buildup Techniques with Short Fiber-Reinforced Composite and Polyethylene Fiber: In Vitro Evaluation of the Fracture Load and Mode of Failure

**DOI:** 10.3390/ma19163510

**Published:** 2026-08-19

**Authors:** Guilherme Scopel Rodrigues, Larissa Moreira Wohlfeil, Aureane Lopes da Silva, Hélio Radke Bittencourt, Gilberto Antonio Borges, Ana Maria Spohr

**Affiliations:** 1 Department of Restorative Dentistry, School of Dentistry, Pontifical Catholic University of Rio Grande do Sul, Porto Alegre 90619-900, RS, Brazil; scopel.guilherme@edu.pucrs.br (G.S.R.); larissa.moreira002@edu.pucrs.br (L.M.W.); 2 Department of Restorative Dentistry, School of Dentistry, University of Uberaba, Uberaba 38055-500, MG, Brazil; aureanelopesdasilva@gmail.com (A.L.d.S.); gilberto.borges@uniube.br (G.A.B.); 3 Department of Statistics, Polytechnic School, Pontifical Catholic University of Rio Grande do Sul, Porto Alegre 90619-900, RS, Brazil; heliorb@pucrs.br

**Keywords:** biomimetics, composite resin, dental restoration, fracture load

## Abstract

The aim was to evaluate, in vitro, the fracture load of single-rooted teeth restored using post-and-core techniques combining short fiber-reinforced composite (SFRC) and polyethylene fiber (PEF) compared with the conventional technique of post-and-core buildup using fiberglass posts (FGPs). Thirty-six single-rooted anterior teeth (12 mm root length) were assigned to three groups (n = 12): Group 1 (FGP + conventional composite resin), Group 2 (SFRC), and Group 3 (PEF + SFRC). All specimens received composite resin crowns, were subjected to 1,000,000 mechanical cycles (100 N, 45°), and were then tested for fracture resistance. Failure mode was assessed visually. After mechanical cycling, no fractures, chipping, cracks, or restoration displacement were observed in any specimen. According to the Kruskal–Wallis test and Dunn’s post hoc test with Bonferroni correction, Group 2 (946 N) showed the highest fracture load, with no significant difference from Group 3 (820 N) (*p* > 0.05). The lowest median was observed in Group 1 (513 N), which differed significantly from the other two groups (*p* < 0.05). Irreparable failures occurred in Group 1 (75%), Group 2 (50%) and Group 3 (59%). It was concluded that post-and-core technique using SFRC, either alone or in combination with PEF, is a viable and mechanically superior alternative to FGP for rehabilitating single-rooted teeth without a ferrule.

## 1. Introduction

The rehabilitation of endodontically treated anterior teeth (ETATs) with severely compromised coronal tooth structure remains a challenge in restorative dentistry. Endodontically treated teeth exhibit mechanical properties that differ significantly from those of vital teeth, with the primary alterations in tooth biomechanics largely attributable to tissue loss resulting from carious lesions, fractures, or cavity preparation, including the endodontic access cavity [1,2].

There is a consensus in the literature that the presence of a ferrule is the primary factor influencing the fracture resistance of ETATs and leads to more favorable failure patterns [3,4,5,6,7]. However, the partial or complete absence of a ferrule in ETATs is not uncommon. Traditionally, intraradicular posts using different techniques have been employed to restore ETATs. Systematic reviews compared fiberglass posts (FGPs) and metal posts, and no significant difference was observed in terms of success or failure rates between these two posts [8,9]. Furthermore, in ETATs with a ferrule, FGPs do not significantly increase fracture resistance compared with composite resin cores without posts [4,6], and a systematic review concluded that most studies do not confirm a positive effect of post placement [10]. Additionally, new evidence suggests that posts may not be necessary even in severely compromised ETATs without a ferrule, as they significantly increase the risk of irreparable failures [6], as well as the risk of deviation or perforation due to additional wear of canal walls [11]. When post-and-core buildups without posts or even endocrowns are used, the risk of catastrophic failure is reduced [12,13,14].

The introduction of novel materials and minimally invasive dentistry has shifted toward the investigation of approaches without posts as a biomimetic alternative using composite resin post-and-core restorations for endodontically treated teeth. Composite resin post-and-core can be made with conventional or bulk-fill composite resins [14]. However, short fiber–reinforced composites (SFRCs) are more recent materials, with the potential to act as load-bearing support barriers under high occlusal forces [15].

The initial commercial presentation of SFRC was launched in 2013 as a bulk-fill material (EverX Posterior, GC, Tokyo, Japan). More recently, in 2019, a flowable material (EverX Flow, GC) was introduced. Both versions consist of a resin matrix and inorganic particles, into which randomly oriented glass microfibers are incorporated. The main variation among these materials lies in the length and diameter of the fibers. EverX Posterior contains longer fibers (1300–2000 μm) with a larger diameter (17 µm), whereas EverX Flow has shorter fibers (200–300 μm) and a smaller diameter (6 µm) [15].

The mechanism of SFRCs is based on the transfer of stress from the polymeric resin matrix to the fibers. SFRCs are indicated for use in the internal layers of restorations and serve as a substitute for dentin [16]. Promising results have recently been reported for the use of SFRCs in endodontically treated posterior teeth. Significant improvements in mechanical properties—such as fracture resistance and failure mode [17,18,19,20,21], fatigue resistance and crack propagation tendency [22,23,24], as well as survival rate [25]—have been observed. However, the use of SFRCs as post-and-core materials to restore severely damaged ETATs has recently emerged. Despite the limited number of existing studies addressing this topic, the results appear promising. One study revealed that the material used to make the post-and-core buildup (conventional resin composite, bulk-fill resin composite, or SFRC) does not significantly influence the survival or failure mode of ETATs without a ferrule and restored with bonded ceramic crowns [14]. However, the authors stated that SFRC seems to be a promising option, since the post-and-core of SFRC was the only one that showed superior survival (cycles endured) in comparison with the group restored with FGP. Additionally, a recent meta-analysis revealed only two studies that investigated the fracture resistance and failure mode of ETATs restored with SFRC as a post-and-core material in comparison to conventional post types [26]. This meta-analysis concluded that there is limited evidence on the laboratory fracture and failure performance of SFRC as post-and-core restorations, and additional studies are necessary [26].

Polyethylene fibers (PEFs) (Ribbond fibers) are older than SFRCs. They consist of a translucent leno-woven structure made from flexible polyethylene fibers with an ultrahigh elastic modulus [27]. PEFs strongly adhere to dental composites as a result of cold gas plasma treatment [28], which allows thorough resin penetration into the fibers, preventing void formation and making Ribbond a durable and integral component of fiber-reinforced composite restorations [29]. The lock-stitch configuration of Ribbond’s leno weave enables efficient distribution of forces throughout the fiber network without transmitting stress to the resin matrix [30]. In this way, PEFs have been used as a core to restore ETATs, demonstrating significant improvement in fracture resistance [1,27,31] and good clinical performance after 2.5 years in endodontically treated primary anterior teeth [32] and after three years in endodontically treated permanent anterior teeth [33].

In light of the positive results obtained with the use of SFRCs and PEFs, two in vitro studies have evaluated the combination of these materials for restoring endodontically treated premolars and have shown higher fracture resistance in comparison to groups in which only posts were used [34,35]. However, to the best of the authors’ knowledge, no study has investigated the combined use of SFRCs and PEFs as post-and-core materials to restore ETATs without a ferrule, particularly with regard to the intrinsic strength of the final restoration. Therefore, the aim of this study was to evaluate, in vitro, the fracture load of ETATs restored using post-and-core buildups with SFRC and PEF combined with SFRC compared with the conventional technique using FGP and a core with conventional composite resin. The study was conducted under the research hypotheses that there are significant differences in (1) fracture load and (2) failure mode among the post-and-core buildup techniques tested in the present study.

## 2. Materials and Methods

### 2.1. Ethical Aspects and Sample Size Calculation

The research was approved by a local Ethics Committee (84608824.0.0000.5336) on 6 December 2024. The sample size calculation considered three groups (factor levels = 3), a significance level (α) of 5%, a power of 80%, and an assumed standard deviation of 300 N according to a pilot study. On the basis of the expected maximum difference of 400 N between groups, the required sample size was 12 specimens per group, for a total of 36 teeth.

### 2.2. Selection of the Teeth

Single-rooted anterior teeth were obtained through the signing of the informed consent form for tooth donation. The teeth were cleaned and immersed in 0.5% chloramine T solution for 24 h for disinfection and then stored in distilled water at 4 °C. To standardize the size of the teeth, a digital caliper (Mitutoyo, Aurora, IL, USA) was used to measure the mesiodistal and buccal–palatal dimensions of the root. Afterward, 36 teeth with similar root proportions and root canal dimensions were selected, including 3 maxillary premolars, 9 mandibular premolars, 13 maxillary central incisors, and 11 maxillary lateral incisors. The mean buccolingual root dimension at the cervical region was 6.3 ± 0.8 mm, and the mean mesiodistal root dimension was 4.4 ± 0.5 mm.

The crowns were removed using a double-sided diamond disc (Extec, London, UK) mounted on a metallographic cutting machine (Extec, London, UK) under constant water irrigation, resulting in roots measuring 12 mm in length. The roots were randomly assigned to one control group and two experimental groups (n = 12).

### 2.3. Endodontic Treatment

Chemomechanical endodontic treatment was performed according to the following protocol: the canals were instrumented up to size 40 using hand files (Sybron Endo, Orange, CA, USA) at working length and irrigated with 2.5% NaOCl at each instrument change, followed by the application of trisodium EDTA for 3 min and subsequent rinsing with saline solution (Biodinâmica, Ibiporã, PR, Brazil). Gutta-percha cones (Tanari, Salvador, BA, Brazil) were coated with Endofill sealer (Dentsply Sirona, Charlotte, NC, USA) and used for obturation by means of the lateral condensation technique. Finally, a 1 mm-thick barrier of glass ionomer cement Gold Label 2 LC (GC, Tokyo, Japan) was placed.

### 2.4. Tooth Embedding and Specimen Preparation

A 10 mm-long root was embedded in self-cured acrylic resin using a plastic cylinder measuring 20 mm in diameter and 20 mm in height. The roots were randomly assigned to one of three groups as described below.

**Figure 1:**
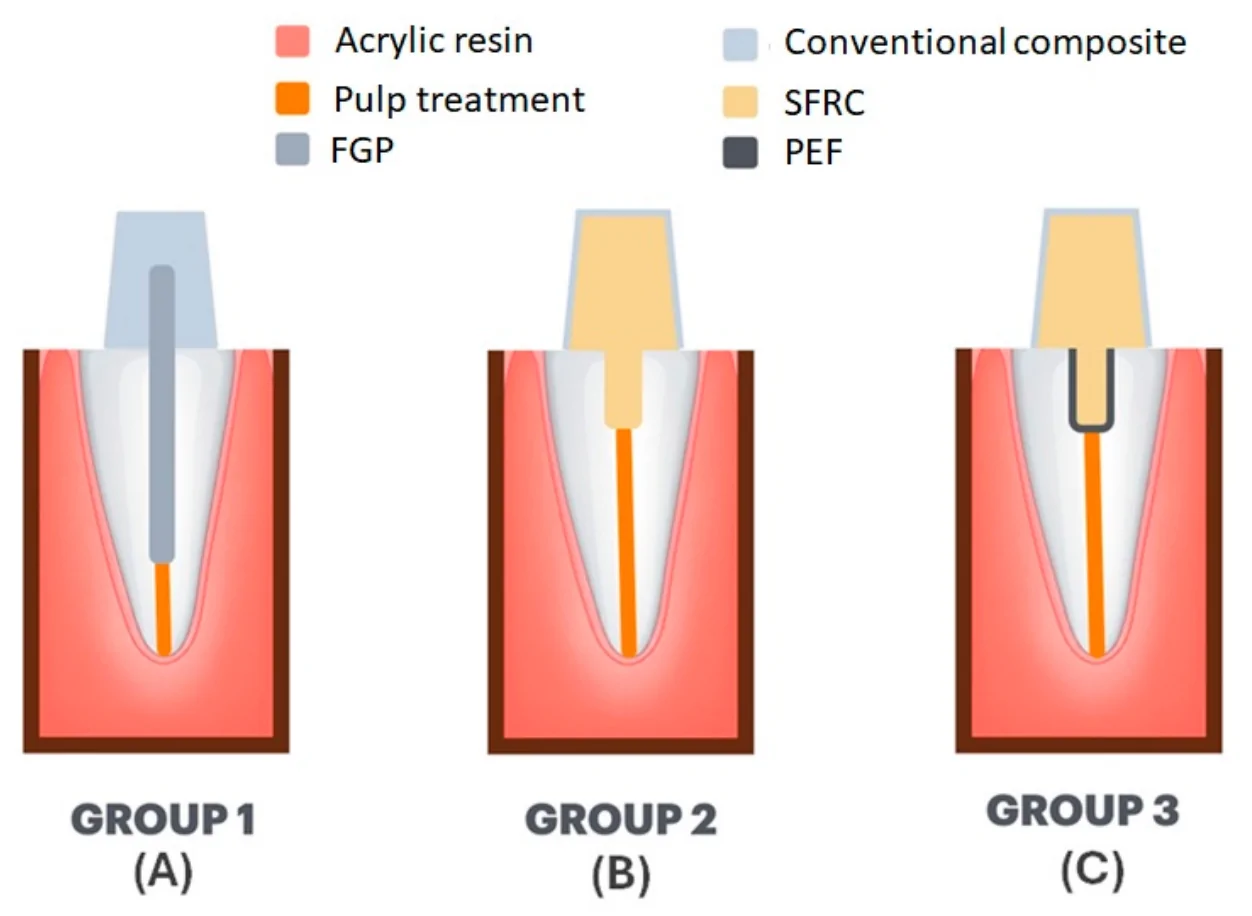
Illustration of the three post-and-core techniques: (**A**) Group 1, using an FGP and a composite resin core; (**B**) Group 2, using SFRC; and (**C**) Group 3, using a PEF and SFRC. All specimens were restored with composite resin crowns.

Group 1 (control): Approximately two-thirds of the root filling material (8 mm) was removed using a size 2 Largo bur (MK Life, Porto Alegre, RS, Brazil). The surface was sandblasted with 50 µm aluminum oxide (Al_2_O_3_) particles (Bioart, Florianópolis, SC, Brazil) for 5 s at a 120° angle and a distance of 1 cm, followed by air–water rinsing for 10 s and drying for 30 s (Figure 1A). The FGP (Angelus, Londrina, PR, Brazil) was treated with silane (Angelus, Londrina, PR, Brazil) and cemented into the root canal using the self-adhesive resin cement RelyX U200 (Solventum, Saint Paul, MN, USA). The resin cement was light-cured using a Valo Grand Cordless unit (Ultradent, South Jordan, UT, USA) for 12 s in high-power mode, with a light output of 1600 mW/cm^2^. The same light-curing unit and output were used for all procedures. After cementation, a 5 mm-high core buildup was fabricated above the cervical margin using Estelite Posterior composite resin (Tokuyama, Tokyo, Japan). Composite resin increments of 2 mm were applied, and each increment was light-cured for 12 s. The FGP was cut using a 4138 diamond bur (KG Sorensen, Serra, ES, Brazil).

Group 2 (EverX Flow): Approximately one-third of the root filling material (4 mm) was removed using a size 2 Largo bur. The surface was sandblasted with 50 µm aluminum oxide (Al_2_O_3_) particles for 5 s at a 120° angle and a distance of 1 cm, followed by air–water rinsing for 10 s and drying for 30 s. The primer of the Clearfil SE Bond adhesive system (Kuraray, Osaka, Japan) was actively applied for 20 s, followed by a gentle air stream for 30 s. Subsequently, a layer of the adhesive was applied and light-cured for 12 s. A post-and-core buildup with a total height of 9 mm was then fabricated using EverX Flow composite resin (GC, Tokyo, Japan) (4 mm intraradicular and 5 mm above the cervical margin). Composite resin increments of 2 mm were applied, and each increment was light-cured for 12 s. The core was then covered with a thin layer of G-aenial Universal Flow composite resin (GC, Tokyo, Japan) to prevent fiber exposure and subsequently light-cured (Figure 1B).

**Figure 2:**
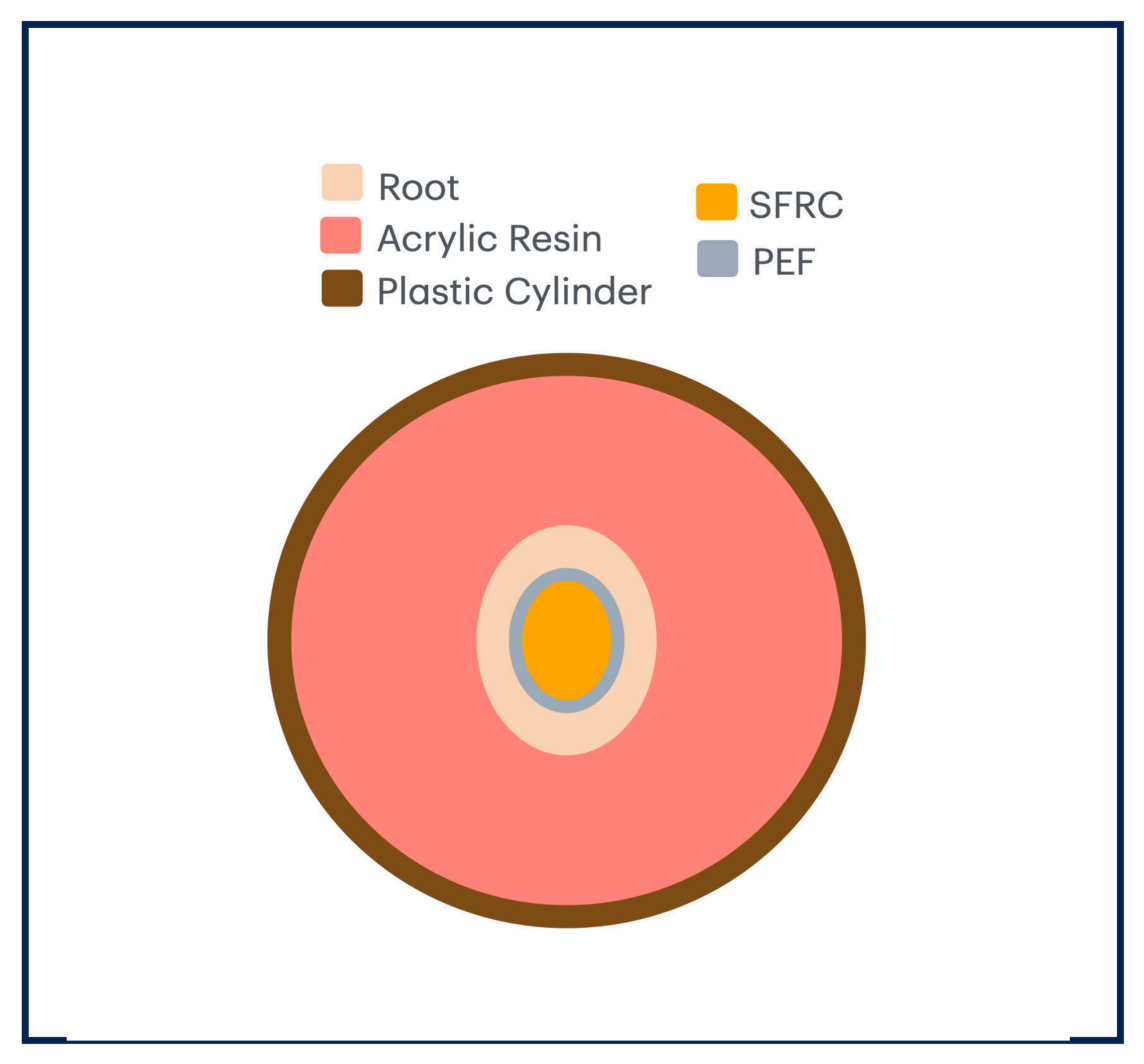
Schematic illustration of Group 3. Transverse view of the combined application of PEF and SFRC.

Group 3 (Ribbond + EverX Flow): Approximately one-third of the root filling material (4 mm) was removed using a size 2 Largo bur. The surface was sandblasted with 50-µm aluminum oxide (Al_2_O_3_) particles for 5 s at a 120° angle and a distance of 1 cm, followed by air–water rinsing for 10 s and drying for 30 s. The self-etch adhesive system Clearfil SE Bond was applied as described for Group 2. Subsequently, a 3 mm-wide PEF (Ribbond, Seattle, WA, USA) piece was wetted with G-aenial Universal Flow composite resin, and excess resin was removed using a microbrush. The PEF was then positioned along the root canal walls using the “wallpapering” technique with the aid of a spatula (Condensa LMArte, Quinelato, Rio Claro, SP, Brazil), followed by light-curing for 12 s. Subsequently, the post-and-core buildup was performed using EverX Flow composite resin (4 mm intraradicular and 5 mm above the cervical margin). Composite resin increments of 2 mm were applied, and each increment was light-cured for 12 s. The core was then covered with a thin layer of G-aenial Universal Flow composite resin (GC, Tokyo, Japan) to prevent fiber exposure and subsequently light-cured (Figure 1C and Figure 2).

In the three groups, the final procedure involved the application of a hydrosoluble gel barrier (KY Jelly, Johnson & Johnson, New Brunswick, NJ, USA) over the core, and an additional light-curing step for 12 s was performed to eliminate the oxygen-inhibited layer.

### 2.5. Crown Fabrication and Cementation

Full crowns of Gradia Direct composite resin (GC, Tokyo, Japan) were fabricated in a standardized manner. The crowns were made with a silicone model based on a copy of artificial teeth, with dimensions of 8 mm × 11 mm. The internal surface of the crowns was sandblasted with Al_2_O_3_ particles (50 µm) for 5 s, followed by air–water rinsing for 10 s and surface drying for 30 s. Only the adhesive bond component of Clearfil SE Bond was applied to the internal surface of the crown using a microbrush, and excess material was removed with another microbrush. The surface of the cores was also sandblasted with Al_2_O_3_ particles (50 µm) for 5 s, followed by rinsing with air–water for 10 s and drying for 30 s. Only the adhesive bond component of Clearfil SE Bond was applied to the cores using a microbrush, and the excess was removed with another microbrush. Gradia Direct composite resin, preheated to 60 °C using a Calset (AdDent, Danbury, CT, USA), was inserted into the crown, which was then positioned over the core and manually pressed until complete seating and material excess extrusion occurred. Excess material was removed using spatulas and brushes, followed by light-curing with a Valo Grand Cordless light-curing unit for 12 s on each surface (mesial, buccal, distal, palatal, and incisal), totaling 60 s, in high-power mode with an output of 1600 mW/cm^2^. All the samples were stored in distilled water at 37 °C for 24 h.

### 2.6. Cycling Mechanical Loading

After storage, the samples were aged in a mechanical loading machine (ER-11000, Erios, São Paulo, SP, Brazil) using the permanent contact mode. The machine piston was positioned on the middle third of the palatal surface of the restoration, applying the load at a 45° angle to the long axis of the tooth. The samples were kept in water at 37 °C, and a load of 100 N and a frequency of 1 Hz were applied for 1,000,000 cycles. At the end of the mechanical cycling, the presence of cracks, chipping, fractures, or restoration displacement was evaluated using a magnifying lens with 10× magnification. The following classifications were assigned: (a) success (no changes); (b) failure (fracture, chipping, cracks, or restoration displacement); and (c) survival (some type of failure without interfering with the use of the restoration).

### 2.7. Fracture Load Testing

The fracture load test was performed using a universal testing machine (Instron 34TM-10, São José dos Pinhais, PR, Brazil) equipped with a 10 kN load cell and a crosshead speed of 1 mm/min. A 6 mm-diameter metal sphere was screwed onto the movable arm of the testing machine, where the load cell was attached. The specimen was positioned on a metal platform previously fixed to the lower base of the machine. A compressive load was applied at a 45-degree oblique angle with a single contact on the palatal surface. The maximum load in Newtons (N) was recorded at the point of fracture.

### 2.8. Failure Analysis

After the fracture load test, the specimens were visually examined to determine the type of failure, which was classified as: (1) repairable (loss of adhesion between the FGP and the root, crown fracture, failure at the crown–root interface, or any other failure that preserved the root and allowed a new restorative procedure); or (2) irreparable (longitudinal, horizontal or oblique root fracture that precluded any possibility of a new restorative procedure).

### 2.9. Statistical Analysis

The statistical analysis was carried out using SPSS 17.0 software (SPSS Inc., Chicago, IL, USA). The results obtained from the fracture load test were subjected to the Kolmogorov–Smirnov normality test and Levene’s test for homogeneity of variance. Although the data were normally distributed (*p* > 0.05), the variances were heterogeneous (*p* = 0.0011). Therefore, the Kruskal–Wallis test and Dunn’s test with Bonferroni correction were used. The chi-square test analyzed the failures. The level of significance was set at 5%.

## 3. Results

After mechanical cycling, none of the specimens exhibited any type of failure, and all the specimens were classified as “success”.

**Figure 3:**
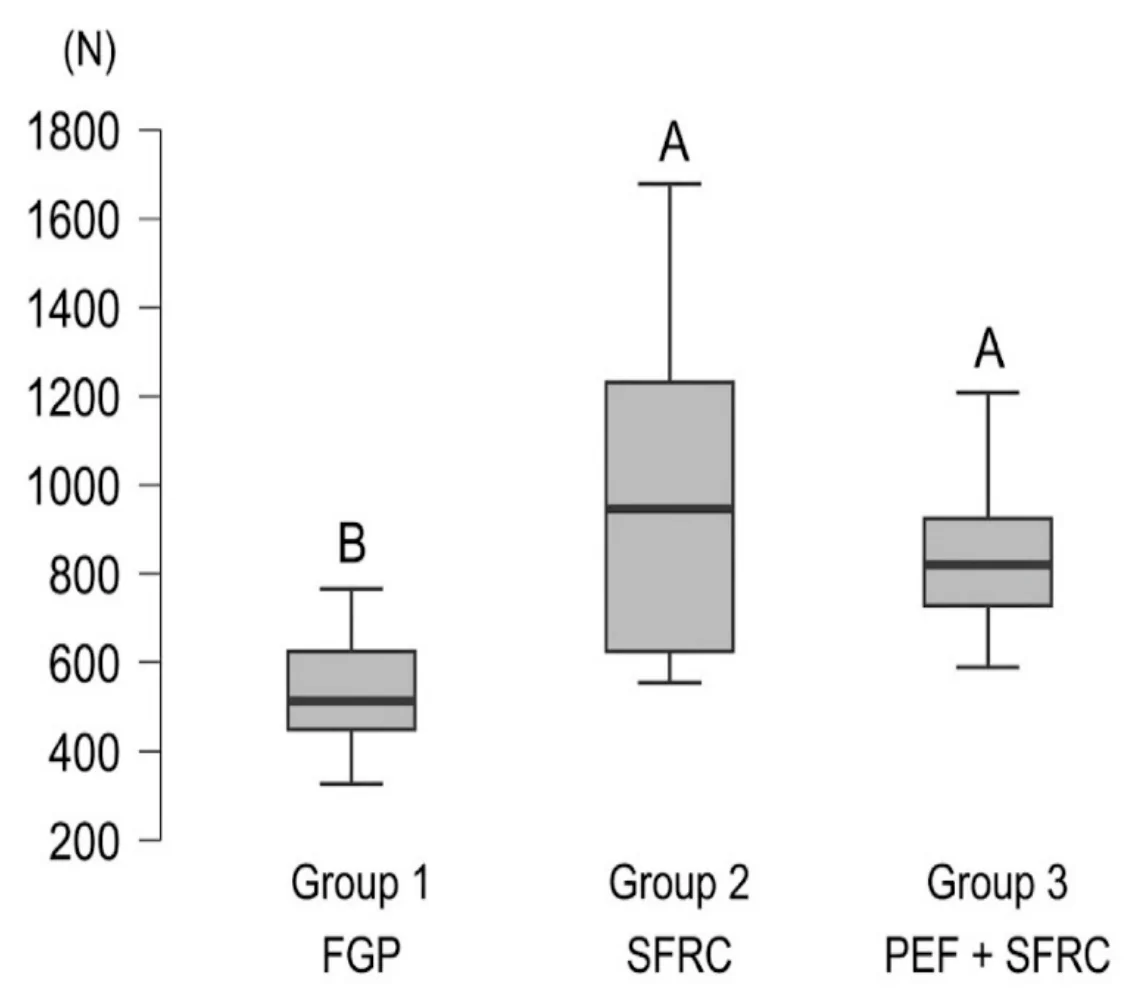
Fracture load (N) of the different groups. Box plots represent the median and interquartile range (IQR; 25th–75th percentiles), with whiskers extending from the minimum to the maximum values. Different letters indicate statistically significant differences according to Dunn’s post hoc test with Bonferroni correction (*p* < 0.05).

The results of the Kruskal–Wallis test revealed a statistically significant difference among the groups (*p* = 0.0005). According to Dunn’s post hoc test with Bonferroni correction, Group 2 (946 N) exhibited the highest fracture load, with no significant difference compared to Group 3 (820 N) (*p* > 0.05). The lowest fracture load was obtained in Group 1 (513 N), which differed significantly from the other two groups (*p* < 0.05) (Table 1 and Figure 3).

**Figure 4:**
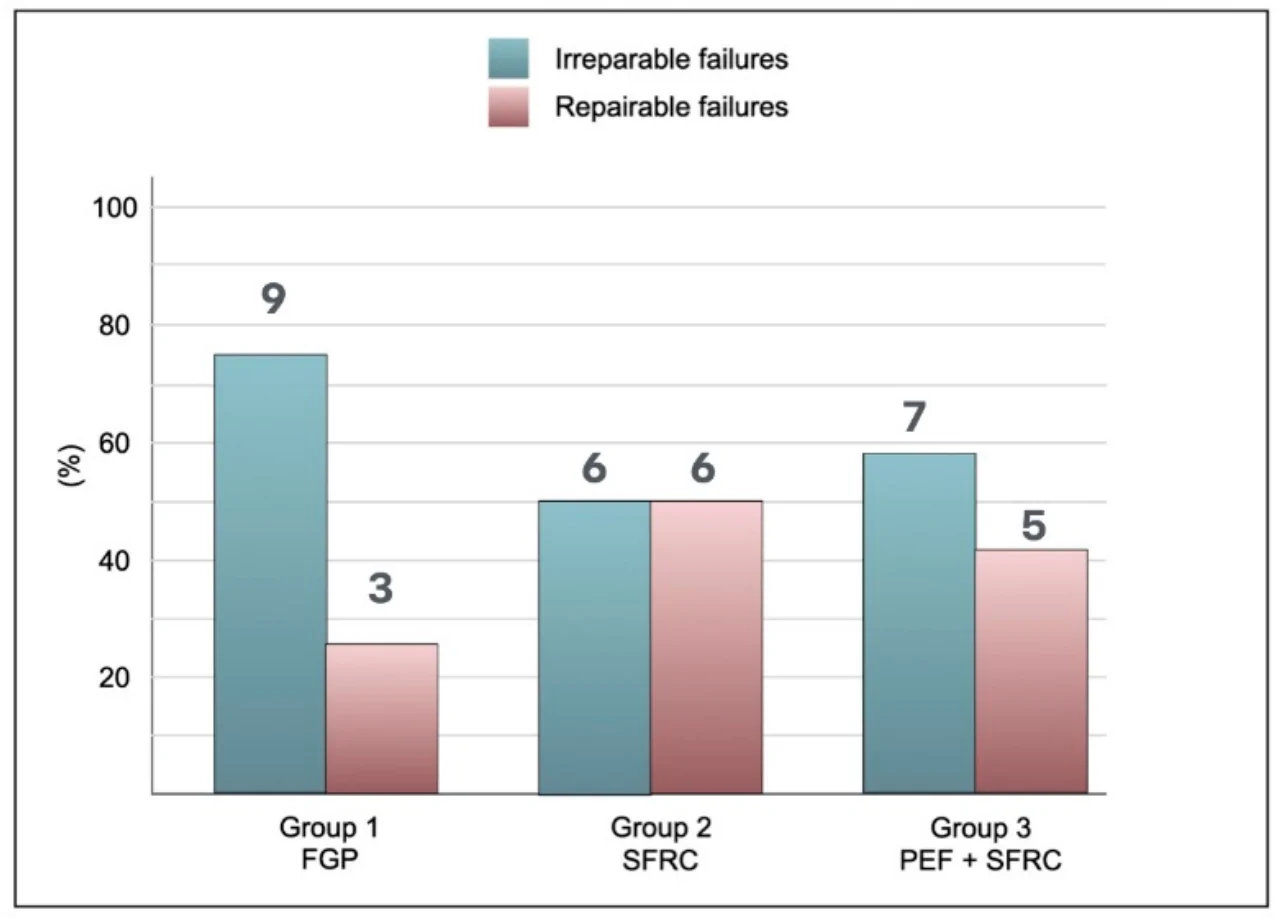
Failure analysis of the different groups. Numbers above the bars indicate the number of specimens in each category.

**Figure 5:**
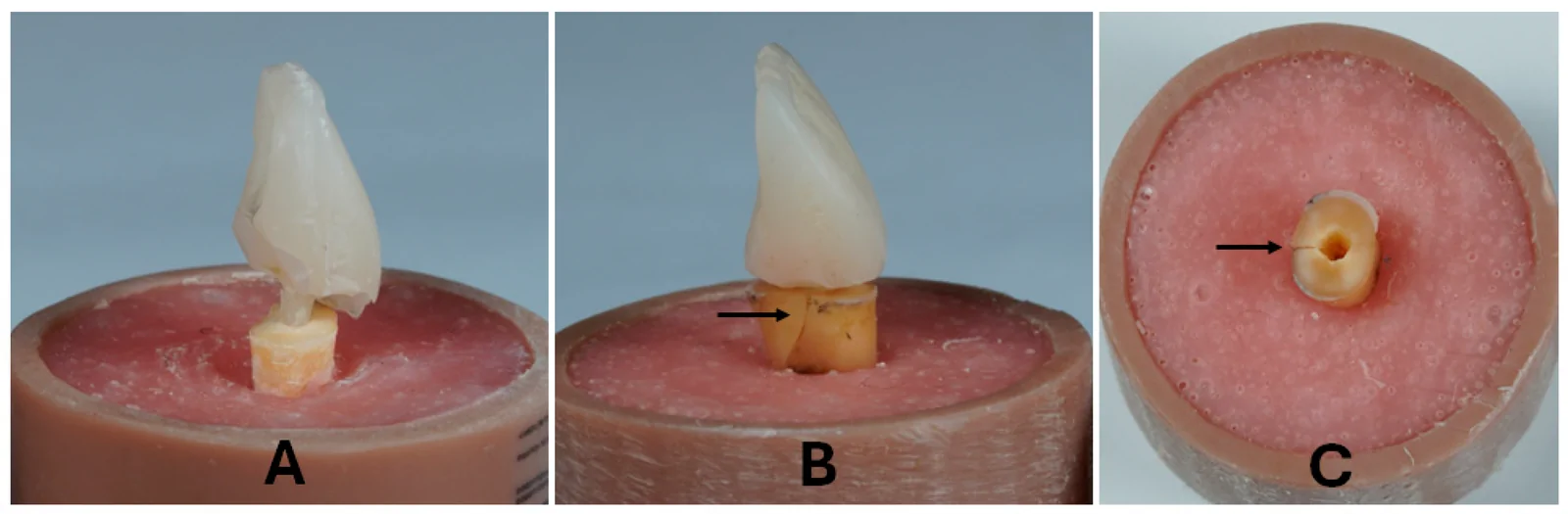
Failures in Group 1: (**A**) repairable failure (failure between the crown and the root, preserving the root); (**B**,**C**) irreparable failures (longitudinal fracture in the root). The arrow indicates a longitudinal root fracture.

**Figure 6:**
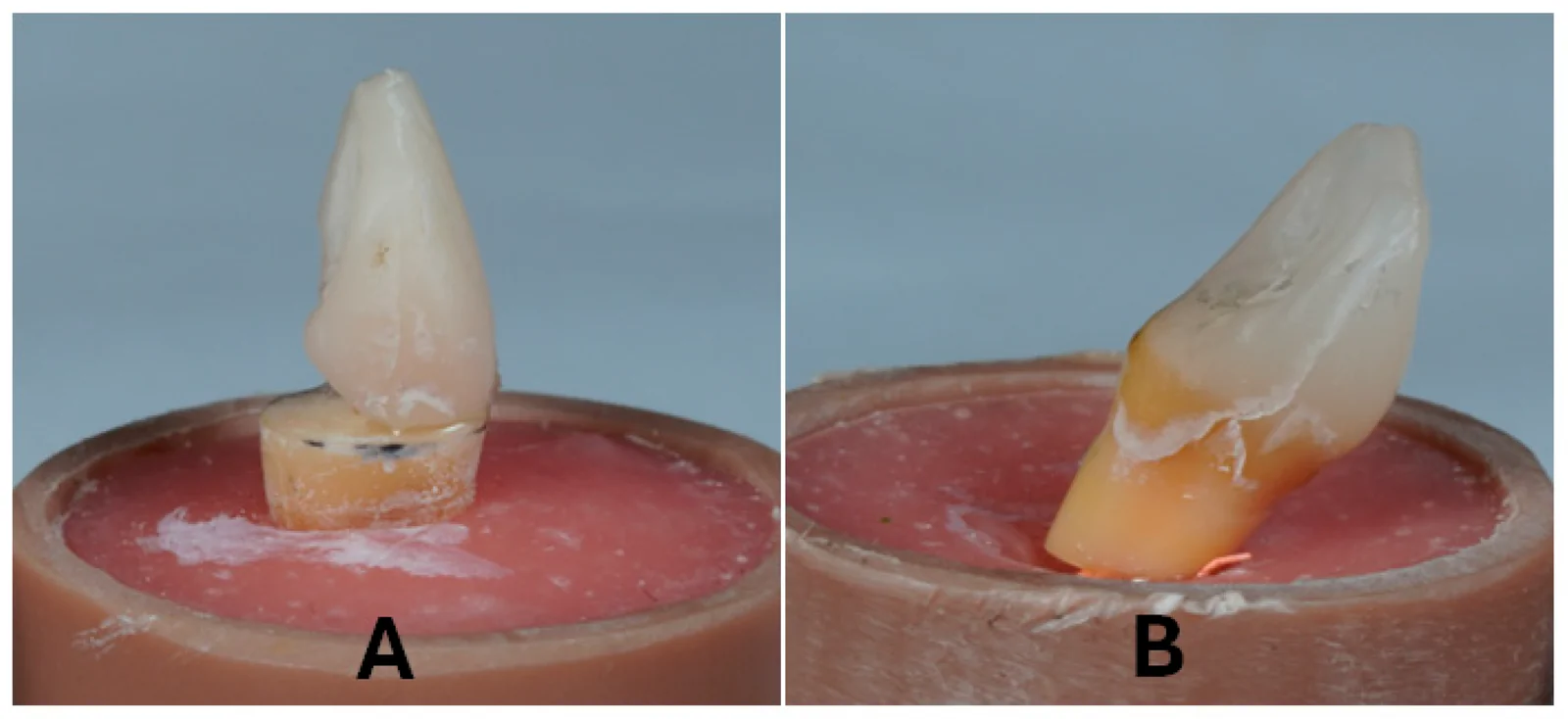
Failures observed in Groups 2 and 3: (**A**) repairable failure between the root and the restoration, preserving the root; (**B**) irreparable failure characterized by horizontal root fracture.

According to the Chi-square test, the distribution of reparable and irreparable failures did not differ significantly among the three groups (*p* = 0.441). Group 1 showed a predominance of irreparable failures (75%). Group 2 presented 50% repairable failures and 50% irreparable failures. Group 3 exhibited 41% repairable failures and 59% irreparable failures (Figure 4). Figure 5 and Figure 6 illustrate the failures observed in the study.

## 4. Discussion

The present study reproduced the restoration of endodontically treated anterior teeth with extensive coronal destruction and no ferrule, representing the most challenging rehabilitative scenario. In these cases, the use of FGP cemented within the root canal has been the conventional technique for core build-up [1,3,5]. Thus, the use of FGP was considered the control group (Group 1) in the present study. Comparisons were made with SFRC post-and-core buildup (Group 2) and PEF combined with SFRC post-and-core buildup (Group 3). Over the cores, a standardized composite resin full crown was cemented, simulating the real clinical situation.

Initially, all the samples were subjected to 1,000,000 mechanical loading cycles with a 100 N load. This cycling regimen simulated approximately four years of natural function, since 250,000 cycles corresponded to one year of average mastication [36]. During mechanical cycling, the load was applied to the middle third of the palatal surface of the restoration at a 45° angle in water at 37 °C to simulate oral conditions. After mechanical cycling, no failures, such as cracks, fractures, or restoration displacement, were observed in any specimen, indicating positive outcomes for all restorative techniques. All the samples were subsequently subjected to a fracture load test and failure mode analysis. According to the results, significant differences in fracture load were observed, whereas no significant differences were found in the distribution of reparable and irreparable failures among the groups. Thus, the first research hypothesis was accepted, and the second research hypothesis was rejected.

The lowest fracture load was obtained in Group 1 (513 N), in which the FGP was used, and this group experienced the greatest percentage of irreparable failures. Despite the absence of a significant difference in failure mode, this group exhibited a tendency toward a higher percentage of irreparable failures than those restored with SFRC and PE. This finding is consistent with other studies [4,12,13] and is related to stress concentration at the interface between the FGP and the resin cement [37]. Although FGP has an elastic modulus closer to that of dentin, the adhesive interface remains the weakest link. When the micromechanical interlocking and the adhesion fail, the masticatory load becomes concentrated at the interface, reducing the strength of the assembly [37]. In anterior teeth without a ferrule, a load applied at 45 degrees generates bending forces. The post, being a solid and prefabricated structure, is unable to dissipate the forces efficiently; instead of distributing the force throughout the entire root structure, the post acts as an internal wedge, directing stress toward the thinner dentinal walls [12,38]. Furthermore, the presence of multiple adhesive interfaces—between the post and the cement and between the cement and dentin—increases the likelihood of cohesive or adhesive failures under cyclic fatigue [39]. Studies have emphasized that the relative stiffness of the FGP accelerates the degradation process of the hybrid layer within the root canal when the tooth is subjected to constant oblique loads [4,38]. Furthermore, the rigidity of the FGP generates stress peaks at the adhesive interface in the absence of a ferrule, facilitating adhesive failure of the structure [38]. Although mechanical cycling for 1,000,000 cycles under a 100 N load did not cause failure in the samples, it was associated with stress at the adhesive interfaces and degradation processes combined with the internal wedge effect [40].

Group 2, in which the SFRC was used, had the highest fracture load value (946 N), which differed significantly from that of the control group. In addition, half of the failures were considered repairable. These data suggest that the biomechanical behavior of ETATs without a ferrule may be more favorable when the root canal is volumetrically filled with SFRC than when it is filled with an FGP, which is a rigid body inserted into the root canal. Therefore, the restorative technique used in Group 2 may have favored the formation of a resilient monoblock, resulting in a biomechanically efficient structure. These findings corroborate those of another study [14]. In the present study, EverX Flow was used because of its lower viscosity, which facilitated the insertion of the material into the root canal.

The effectiveness of SFRC lies in its high fracture toughness. Randomly dispersed fibers act as physical barriers that dissipate energy and interrupt the propagation of microcracks before they reach critical dimensions [41]. Therefore, in extensively destroyed ETATs, SFRC acts as a material intended to mimic the load-absorbing behavior of natural dentin, promoting higher fracture load values compared with the use of FGP (Group 1). The mechanical properties of SFRC are influenced by the resin matrix composition and fiber concentration. EverX Flow contains flowable monomers such as UDMA and TEGDMA in its composition, which may lead to improved fiber wetting, enhancing their integration with the resin matrix and, consequently, stress dissipation [41,42]. A more homogeneous stress transfer from the resin matrix to the inorganic particles and fibers may increase the flexural strength of the SFRC. In addition, the stress distribution in these composites differs from that in conventional particulate-filled materials. The fibers act as reinforcements, creating connections between different regions of the material that modulate stresses and reduce crack propagation. Therefore, stress transfer from the polymer matrix to the filler particles is an important factor influencing flexural strength values and chain mobility during the polymerization process, affecting the degree of conversion and the material’s mechanical properties [42,43].

The stress-distribution capability of SFRC relies on specific microscopic mechanisms. The fibers within the resin matrix are randomly oriented in three dimensions and have a length exceeding the critical fiber length required for effective load transfer. When stress concentrates at a defect, propagating cracks encounter these randomly oriented fibers. Rather than propagating straight through the material, the crack path undergoes deflection, branching, and energy dissipation. The primary toughening mechanisms involve fiber bridging and fiber pull-out at the fracture zone. As the crack propagates, intact fibers bridge the crack faces behind the crack tip, carrying tensile loads and impeding further crack propagation. When the local shear stress exceeds the fiber–matrix interfacial bond strength, the fibers are pulled out of the polymer matrix rather than fracturing catastrophically. This mechanism dissipates energy and helps prevent sudden structural failure under high mechanical loads [16,41].

Group 3, in which PEF and SFRC were combined, had an intermediate fracture load value (820 N) and fewer irreparable fractures. Different techniques have been used to insert the PEF into the root canal: (a) packing it into the canal space as tightly as possible [33]; (b) insertion of two pieces of PEF shaped into a “V”, one in a facio-lingual direction and the other in a mesio-distal direction inside the root [27]; (c) two portions of PEF positioned parallel to each other, one against the palatal wall and the other against the buccal wall of the cervical portion of the canal [6]; (d) placing the PEF in a “U” pattern, from the palatal wall to the buccal wall; and (e) placing the PEF in a circular pattern, extending through the mesial, lingual, distal, and buccal surfaces of the root [31], also known as the “wallpapering” technique. In the present study, the “wallpapering” technique using PEF was employed. This technique allowed space to remain within the root canal for insertion of the SFRC, enabling the association of both materials within the root canal.

The inclusion of PEF aims to create a supportive network to distribute multidirectional stresses, such as tensile and shear forces, simulating an artificial dentin–enamel junction [44,45]. However, the fracture load in Group 3 did not differ significantly from that of Group 2, and the lower value may have been associated with the insertion of multiple materials into a cavity with reduced dimensions. The incorporation of PEF introduces multiple adhesive interfaces among the dentin, the luting agent, the PEF, and the surrounding SFRC. Each additional interface represents a potential site for microvoid formation, leading to stress concentration and defect initiation under mechanical loading [39]. Since SFRC alone effectively distributes mechanical stresses [16,24], the additional interfaces created by the PEF did not provide additional reinforcement and may have offset any potential strengthening effect. Additionally, the insertion of PEF in a restricted-access environment such as the root canal is challenging, as the canal of an anterior tooth is naturally narrow. Then, the insertion technique of reinforced materials, especially PEF in Group 3, requires a learning curve and manual dexterity that may vary among operators. Nevertheless, Group 3 outperformed the control group, suggesting that the association of fiber-based systems, such as PEF and SFRC, is more resilient than the use of FGP.

The analysis of failure modes is clinically relevant, as the failure pattern may indicate the prognosis of the tooth. Due to the difficulty in obtaining anterior teeth, this study was conducted using the minimum number of teeth, based on the sample size determined for the fracture load test. Therefore, a possible reason for the absence of a significant difference in failure mode is the small sample size, which is considered a limitation of the study. However, the use of FGP (Group 1) exhibited the highest percentage of irreparable fractures, which may indicate a clinically relevant trend that warrants investigation in studies with larger sample sizes. Additionally, caution should be exercised when extrapolating these findings to clinical practice. Embedding the roots in a rigid medium (acrylic resin) does not simulate the resilience and stress dissipation provided by the periodontal ligament [46,47]. In a clinical scenario, this physiological micromobility could alter the stress distribution at the root adhesive interface, potentially reducing the stress peaks observed during testing. Furthermore, the fracture load test involves the application of a static load to the specimen until fracture, which does not fully replicate clinical conditions. Nevertheless, the results of this mechanical test are important for comparing the fracture loads of different restorative techniques.

In the present study, root-level fractures were observed in some samples. Therefore, the adhesive system and the post-and-core buildup materials withstood the static load, transferring stress to the root dentin. As a consequence, the root reached its plastic limit, and fracture occurred before failure of the restorative materials [9]. This behavior was also observed in some specimens restored with SFRC (Group 2) and PEF associated with SFRC (Group 3). Nevertheless, irreparable failures were more frequent in the teeth restored with FGP, corroborating the findings of a meta-analysis [48].

The present study has limitations inherent to its in vitro design, which does not fully replicate clinical conditions [49]. Another limitation of this study is the slight variation in the crown sectioning level required to obtain roots with a standardized length of 12 mm. In some specimens, the crowns were sectioned at the cemento–enamel junction (CEJ), whereas in others they were sectioned below the CEJ. This slight variation in the sectioning level was unavoidable because of differences in tooth length. However, its potential influence on the mechanical test results cannot be excluded. Additionally, no microscopic fractographic analysis of the fractured specimens was performed. Therefore, the toughening mechanisms discussed, including crack deflection, crack bridging, and stress redistribution by the SFRC, were inferred from previous studies rather than directly confirmed by the fracture surface morphology of the specimens evaluated in this investigation.

The use of materials with mechanical properties more closely resembling those of natural dental tissues, such as SFRC, as an alternative to FGPs in severely compromised ETATs has been shown to be a viable approach from both biomechanical and biological perspectives. Although the present study yielded promising results, further research is needed, including studies with larger sample sizes, combined thermal cycling and mechanical fatigue, microscopic fracture surface analysis, and longitudinal clinical studies to confirm the effectiveness of using SFRC, with or without PEF, for the restoration of ETATs without ferrule.

## 5. Conclusions

Despite the limitations of this in vitro study, the following conclusions can be drawn:

- Post-and-core buildup with short fiber-reinforced composite provided higher fracture load in endodontically treated anterior teeth without ferrules compared with the use of fiberglass posts.
- Post-and-core buildup combining polyethylene fiber and short fiber-reinforced composite did not demonstrate superior fracture load to the use of short fiber-reinforced composite alone.
- The use of short fiber-reinforced composite, alone or combined with polyethylene fiber, tended to reduce the incidence of irreparable failures.

## Figures and Tables

**Table 1:** Fracture load values (N) for the different groups.

Groups	n	Median and [IQR] of Fracture Load (N)	vs. Group 1	vs. Group 2	vs. Group 3
Group 1—FGP (Control)	12	513.5 ^B^ [175.3]	—	0.0017	0.003
Group 2—SFRC	12	946.5 ^A^ [606.3]	0.0017	—	1.000
Group 3—PEF + SFRC	12	820.0 ^A^ [195.5]	0.003	1.000	—

Different letters indicate statistically significant differences according to Dunn’s post hoc test with Bonferroni correction (*p* < 0.05). Pairwise *p*-values in the last three columns are adjusted using the Bonferroni correction. IQR represents interquartile ranges.

## Data Availability

The original contributions presented in the study are included in the article; further inquiries can be directed to the corresponding author.

## References

[1] Dietschi D., Duc O., Krejci I., Sadan A. (2007). Biomechanical considerations for the restoration of endodontically treated teeth: A systematic review of the literature—Part 1. Composition and micro- and macrostructure alterations. Quintessence Int..

[2] Caussin E., Izart M., Ceinos R., Attal J.P., Beres F., François P. (2024). Advanced Material Strategy for Restoring Damaged Endodontically Treated Teeth: A Comprehensive Review. Materials.

[3] Juloski J., Radovic I., Goracci C., Vulicevic Z.R., Ferrari M. (2012). Ferrule effect: A literature review. J. Endod..

[4] Magne P., Lazari P.C., Carvalho M.A., Johnson T., Del Bel Cury A.A. (2017). Ferrule-effect dominates over use of a fiber post when restoring endodontically treated incisors: An in vitro study. Oper. Dent..

[5] Silva-Sousa A.C., Moris I.C.M., Barbosa A.F.S., Silva-Sousa Y.T.C., Sousa-Neto M.D., Pires C.R.F., Gomes E.A. (2020). Effect of restorative treatment with endocrown and ferrule on the mechanical behavior of anterior endodontically treated teeth: An in vitro analysis. J. Mech. Behav. Biomed. Mater..

[6] Albashaireh Z.S.M., Sbeih Y.K. (2024). The effect of ferrule and core material on fracture resistance of endodontically treated anterior teeth restored with ceramic crowns after artificial aging. J. Dent..

[7] Baruah S., Ojah P., Nath S., Jain S., Agnihotri N., Duggal S. (2025). Comparative evaluation of ferrule designs regarding fracture resistance about crowned anterior teeth using prefabricated post and composite core under direct loading: An in vitro study. Bioinformation.

[8] Figueiredo F.E.D., Martins-Filho P.R.S., Faria-e-Silva A.L. (2015). Do metal post retained restorations result in more root fractures than fiber post-retained restorations? A systematic review and meta-analysis. J. Endod..

[9] Tsintsadze N., Margmelashvili-Malament M., Natto Z.S., Ferrari M. (2024). Comparing survival rates of endodontically treated teeth restored either with glass-fiber-reinforced or metal posts: A systematic review and meta-analyses. J. Prosthet. Dent..

[10] Naumann M., Schmitter M., Frankenberger R., Krastl G. (2018). “Ferrule comes first. Post is second!” Fake new and alternative facts? A systematic review. J. Endod..

[11] Jurema A.L., Filgueiras A.T., Santos K.A., Bresciani E., Caneppele T.M. (2022). Effect of intraradicular fiber post on the fracture resistance of endodontically treated and retored anterior teeth: A systematic review and meta-analysis. J. Prosthet. Dent..

[12] Lazari P.C., De Carvalho M.A., Del Bel Cury A.A., Magne P. (2018). Survival of extensively damaged endodontically treated incisors restored with different types of posts-and-core foundation restoration material. J. Prosthet. Dent..

[13] De Carvalho M.A., Lazari-Carvalho P.C., Del Bel Cury A.A., Magne P. (2021). Accelerated fatigue resistance of endodontically treated incisors without ferrule restored with CAD/CAM endocrowns. Int. J. Esthet. Dent..

[14] De Carvalho M.A., Lazari-Carvalho P.C., Del Bel Cury A.A., Magne P. (2024). Fatigue and failure analysis of restored endodontically treated maxillary incisors without a dowel or ferrule. J. Prosthet. Dent..

[15] Alshabib A., Jurado C.A., Tsujimoto A. (2022). Short fiber-reinforced resin-based composites (SFRCs); Current status and future perspectives. Dent. Mater. J..

[16] Garoushi S., Gargoum A., Vallittu P.K., Lassila L. (2018). Short fiber-reinforced composite restorations: A review of the current literature. J. Investig. Clin. Dent..

[17] Bijelic-Donova J., Keulemans F., Vallittu P.K., Lassila L.V.J. (2020). Direct bilayered biomimetic composite restoration: The effect of a cusp-supporting short fiber-reinforced base design on the chewing fracture resistance and failure mode of molars with or without endodontic treatment. J. Mech. Behav. Biomed. Mater..

[18] Garoushi S., Akbaşak-Sungur A.Ö., Erkut S., Vallittu P.K., Uctasli S., Lassila L. (2023). Evaluation of fracture behavior in short fiber-reinforced direct and indirect overlay restorations. Clin. Oral. Investig..

[19] Shah S., Shilpa-Jain D.P., Velmurugan N., Sooriaprakas C., Krithikadatta J. (2020). Performance of fibre reinforced composite as a post-endodontic restoration on different endodontic cavity designs—An in-vitro study. J. Mech. Behav. Biomed. Mater..

[20] Pierce A.L., Kosaraju A., Gedge J.L., Vandewalle K.S. (2025). Fracture resistance and Failure Modes of Cuspal-coverage Restorations Using Fiber-reinforced and Non-fiber-reinforced Materials: An In Vitro Study. J. Contemp. Dent. Pract..

[21] Oksanen V., Bijelic-Donova J., Vallittu P.K., Lassila L., Garoushi S. (2025). Two different short fiber-reinforced resin composites for extensive MOD cavities in premolars and molars. Clin. Oral. Investig..

[22] Fráter M., Sáry T., Molnár J., Braunitzer G., Lassila L., Vallittu P.K., Garoushi S. (2022). Fatigue performance of endodontically treated premolars restored with direct and indirect cuspal coverage restorations utilizing fiber-reinforced cores. Clin. Oral. Investig..

[23] Magne P., Milani T. (2023). Short-fiber Reinforced MOD Restorations of Molars with Severely Undermined Cusps. J. Adhes. Dent..

[24] Bijelic-Donova J., Garoushi S., Lassila L.V., Rocca G.T., Vallittu P.K. (2022). Crack propagation and toughening mechanism of bilayered short-fiber reinforced resin composite structure—Evaluation up to six months storage in water. Dent. Mater. J..

[25] Molnár J., Fráter M., Sáry T., Braunitzer G., Vallittu P.K., Lassila L., Garoushi S. (2022). Fatigue performance of endodontically treated molars restored with different dentin replacement materials. Dent. Mater..

[26] Fousekis E., Lolis A., Marinakis E., Oikonomou E., Foros P., Koletsi D., Eliades G. (2025). Short fiber-reinforced composite resins as post-and-core materials for endodontically treated teeth: A systematic review and meta-analysis of in vitro studies. J. Prosthet. Dent..

[27] Hemalatha H., Sandeep M., Kulkarni S., Yakub S.S. (2009). Evaluation of fracture resistance in simulated immature teeth using resilon and ribbond as root reinforcements—An in vitro study. Dent. Traumatol..

[28] Cobankara F.K., Unlu N., Cetin A.R., Ozkan H.B. (2008). The effect of different restoration techniques on the fracture resistance of endodontically-treated molar. Oper. Dent..

[29] Dyer S.R., Lassila L.V., Jokinen M., Vallittu P.K. (2004). Effect of fiber position and orientation on fracture load of fiber-reinforced composite. Dent. Mater..

[30] Chaudhary V., Shrivastava B., Bhatia H.P., Aggarwal A., Singh A.K., Gupta N. (2012). Multifunctional ribbond—A versatile tool. J. Clin. Pediatr. Dent..

[31] Codas-Duarte D., Pelozo L.L., Mazzi-Chaves J.F., Lopes-Olhè F.C., Sousa-Neto M.D., Souza-Gabriel A.E. (2025). Fracture and crack behavior of weakened incisors restored with fiber posts, polyethylene reinforcement, or 3D-pronted endocrowns. Cureus.

[32] Memarpour M., Shafiei F. (2013). Restoration of primary anterior teeth using intracanal polyethylene fibres and composite: An in vivo study. J. Adhes. Dent..

[33] Ayna B., Celenk S., Atakul F., Uysal E. (2009). Three-year clinical evaluation of endodontically treated anterior teeth restored with a polyethylene fibre-reinforced composite. Aust. Dent. J..

[34] Aslan T., Sagsen B., Er Ö., Ustun Y., Cinar F. (2018). Evaluation of fracture resistance in root canal-treated teeth restored using different techniques. Niger. J. Clin. Pract..

[35] Soto-Cadena S.L., Zavala-Alonso N.V., Cerda-Cristerna B.I., Ortiz-Magdaleno M. (2023). Effect of short fiber-reinforced composite combined with polyethylene fibers on fracture resistance of endodontically treated premolars. J. Prosthet. Dent..

[36] Delong R., Douglas W.H. (1991). An artificial oral environment for testing dental materials. IEEE Trans. Biomed. Eng..

[37] Goracci C., Ferrari M. (2011). Current perspectives on post systems: A literature review. Aust. Dent. J..

[38] Zicari F., Van Meerbeek B., Debels E., Lesaffre E., Naert I. (2011). An up to 3-year controlled clinical trial comparing the outcome of glass fiber posts and composite cores with gold alloy-based posts and cores for the restoration of endodontically treated teeth. J. Prosthodont..

[39] Tay F.R., Pashley D.H. (2007). Monoblocks in root canals: A hypothetical or a tangible goal. J. Endod..

[40] Lima V.P., Machado J.B., Zhang Y., Loomans B.A.C., Moraes R.R. (2022). Laboratory methods to simulate the mechanical degradation of resin composite restorations. Dent. Mater..

[41] Garoushi S., Vallittu P.K., Lassila L.V.J. (2007). Direct restoration of severely damaged incisors using short fiber-reinforced composite resin. J. Dent..

[42] Garoushi S., Säilynoja E., Vallittu P.K., Lassila L.V.J. (2013). Physical properties and depth of cure of a new short fiber reinforced composite. Dent. Mater..

[43] Bijelic-Donova J., Garoushi S., Lassila L.V., Keulemans F., Vallitu P.K. (2016). Mechanical and structural characterization of discontinuous fiber-reinforced dental resin composite. J. Dent..

[44] Deliperi S., Alleman D., Rud D. (2017). Stress-reduced direct composites for the restoration of structurally compromised teeth: Fiber design according to the “Wallpapering” Technique. Oper. Dent..

[45] Ozsevik A.S., Yldirim C., Aydin U., Culha E., Surmelioglu D. (2016). Effect of fibre-reinforced composite on the fracture resistance of endodontically treated teeth. Aust. Endod. J..

[46] Soares C.J., Pizi E.C.G., Fonseca R.B., Martins L.R.M. (2005). Influence of root embedment material and periodontal ligament simulation on fracture resistance tests. Braz. Oral. Res..

[47] Rosentritt M., Behr M., Scharnagl P., Handel G., Kolbeck C. (2011). Influence of resilient support of abutment teeth on fracture resistance of all-ceramic fixed partial dentures: An in vitro study. Int. J. Prosthodont..

[48] Vartak M.A., Hegde V.R., Hedge S.R., Fanibunda U. (2025). Fracture resistance and failure modes of endodontically-treated permanent teeth restored with Ribbond posts vs other post systems: A systematic review and meta-analysis of in vitro studies. Restor. Dent. Endod..

[49] Hardan L., Bourgi R., Kharouf N., Mancino D., Zarow M., Jakubowicz N., Haikel Y., Cuevas-Suárez C.E. (2021). Bond strength of universal adhesives to dentin: A systematic review and meta-analysis. Polymers.

